# P-351. Mastering HIV: The Full Spectrum of Training During ID Fellowship

**DOI:** 10.1093/ofid/ofaf695.569

**Published:** 2026-01-11

**Authors:** Douglas S Kepko, Mohammad Madhee Sobhanie, Susan L Koletar, Ashley Lipps

**Affiliations:** Ohio State University, Columbus, OH; The Ohio State University, Columbus, Ohio; Ohio State University, Columbus, OH; The Ohio State University Wexner Medical Center, Columbus, Ohio

## Abstract

**Background:**

The American College of Graduate Medical Education (ACGME) requires that all Infectious Diseases (ID) Fellowship programs provide an ambulatory experience that includes the care of people living with HIV (PLWH). Data is limited on how best to ensure fellows gain experience across the HIV care continuum. Despite HIV being a manageable chronic illness with majority of patients maintaining long term viral suppression, it is vital that ID fellows gain experience managing more complex patients, including those who are newly diagnosed. At The Ohio State University (OSU), all HIV referrals are reviewed by a core group of ID Faculty and newly diagnosed PLWH are preferentially scheduled to be seen by the ID fellows. The objective of our study is to assess the clinical characteristics and complexity of newly diagnosed PLWH cared for in the ID Fellows clinic.

Demographics

**Figure:**
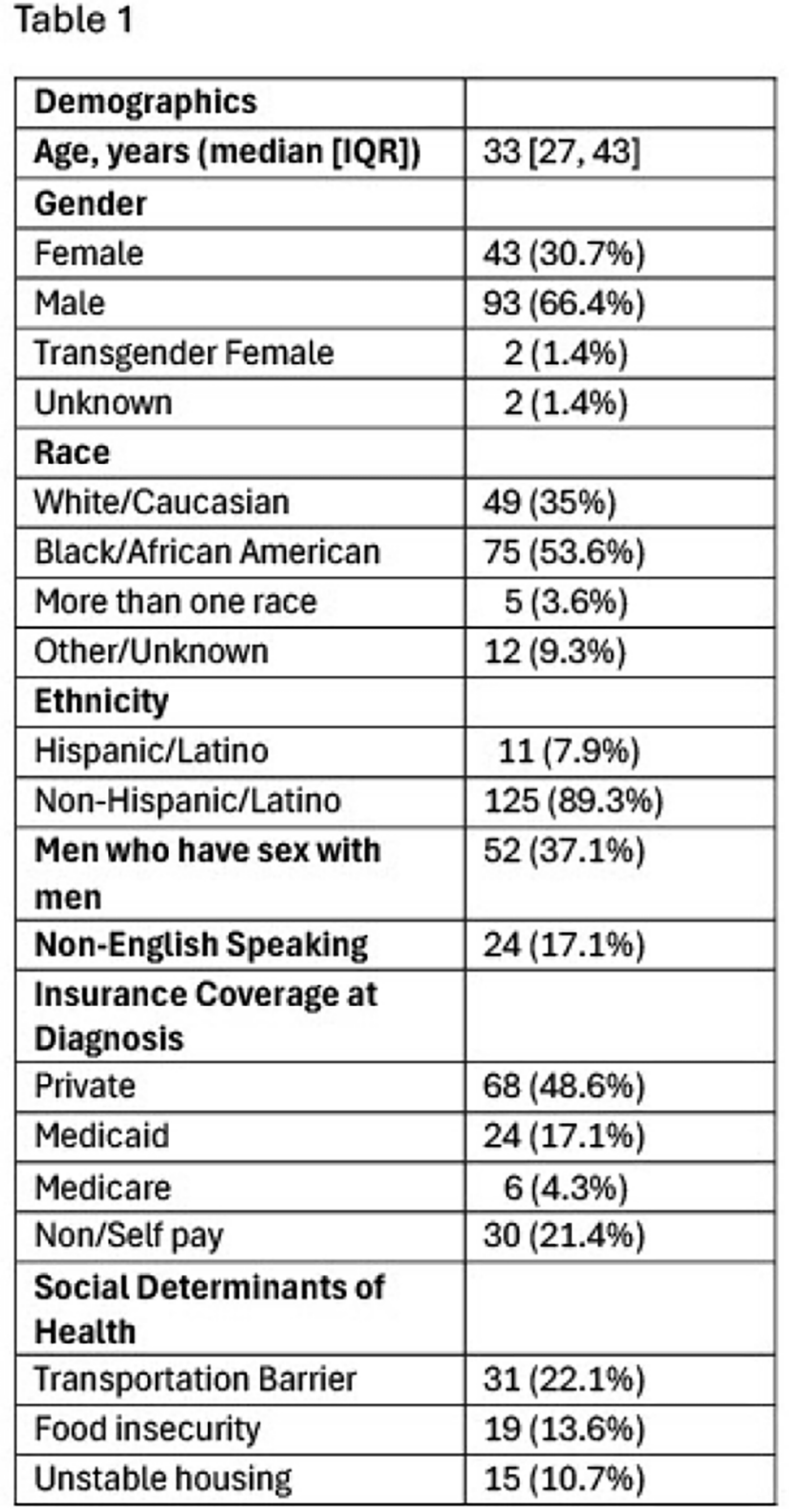

**Figure:**
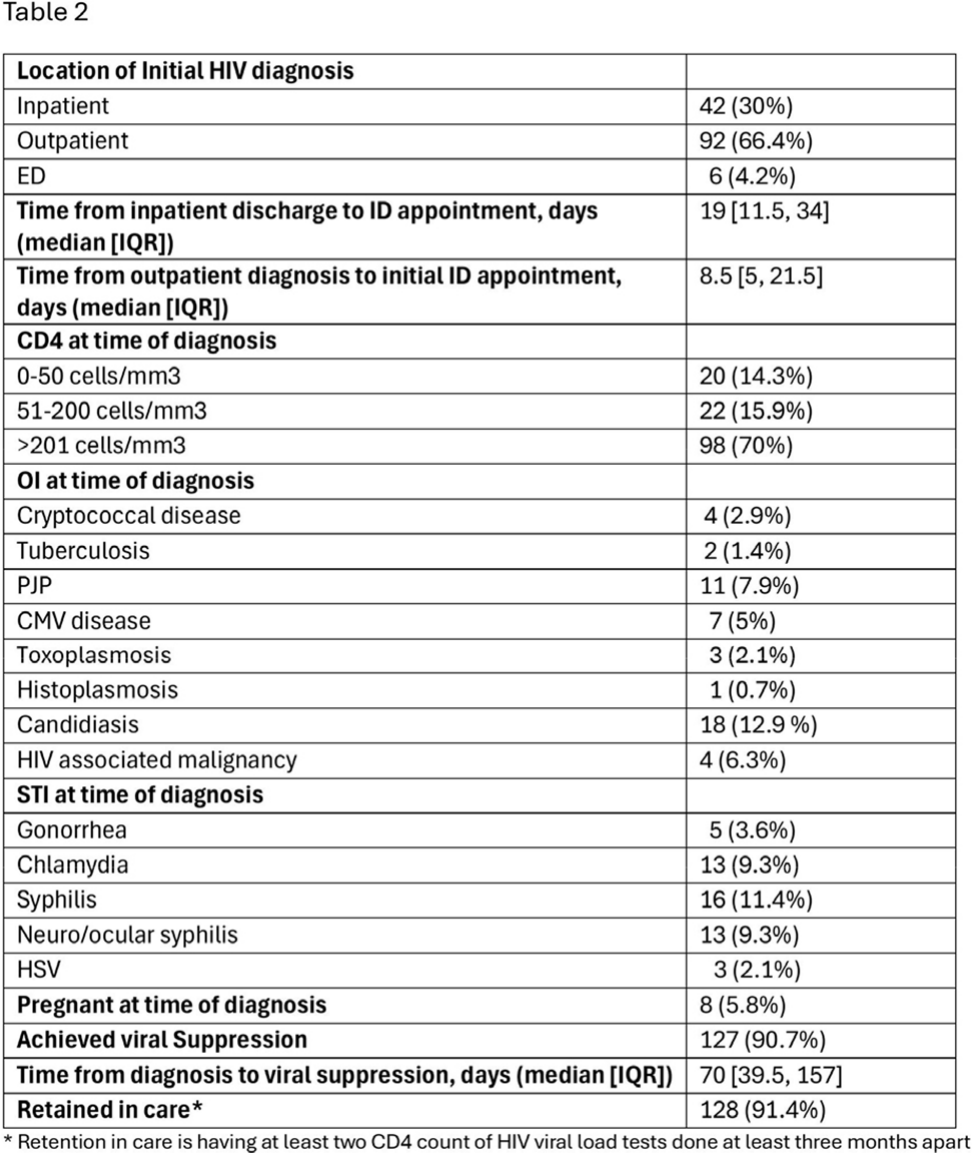

**Methods:**

This was a retrospective study of patients newly diagnosed with HIV who were seen in the OSU ID Fellows Clinic between July 1, 2020 and June 30, 2024. A manual chart review was performed and patient demographics and clinical information were obtained. Categorical variables were reported as counts and percentages and continuous variables were reported as medians and interquartile ranges (IQRs). This study was approved by the OSU Institutional Review Board.

**Results:**

140 newly diagnosed PLWH were seen in ID Fellows Clinic during the study period. Demographic information is shown in Table 1. Clinical outcomes are shown in Table 2. Median time from an outpatient HIV diagnosis to ID appointment was 8.5 [IQR 5, 21.5] days. Forty-two (30.2%) of patients had CD4 count < 200 cells/mm^3^, 50 (36%) had an opportunistic infection or HIV associated malignancy, and 8 (5.8%) were pregnant at time of diagnosis. One hundred twenty-seven (90.7%) achieved viral suppression with a median time to viral suppression of 70 [IQR 39.5, 157] days. One hundred twenty-eight (91.4%) of patients were retained in care.

**Conclusion:**

Targeted scheduling of newly diagnosed PLWH with ID Fellows ensures that fellows gain experience managing diverse and complex patient populations. Despite the various complexities within this population, this process resulted in rapid linkage to care and high rates of viral suppression/retention in care in newly diagnosed PLWH.

**Disclosures:**

Susan L. Koletar, MD, Gilead Sciences: Co-investigator on several grants; no direct salary support|ViiV Healthcare: Grant/Research Support

